# How much online pornography is too much? A comparison of two theoretically distinct assessment scales

**DOI:** 10.1186/s13690-024-01294-5

**Published:** 2024-05-30

**Authors:** Germano Vera Cruz, Elias Aboujaoude, Magdalena Liberacka-Dwojak, Monika Wiłkość-Dębczyńska, Lucien Rochat, Riaz Khan, Yasser Khazaal

**Affiliations:** 1 Department of Psychology, CRP-CPO, University of Picardie Jules Verne, Amiens, France; 2 Department of Psychiatry and Behavioral Sciences, Stanford University School of Medicine, Stanford, CA USA; 3 Program in Internet, Health and Society, Cedars-Sinai Medical Center, Los Angeles, CA USA; 4 Department of Psychology, Kazimierz Wielki University, Bydgoszcz, Poland; 5 Department of Mental Health and Psychiatry, Specialized Facility in Behavioral Addiction ReConnecte, University Hospitals of Geneva, Geneva, Switzerland; 6 Department of Mental Health and Psychiatry, Frontier Medical College Abbottabad, Bahria University Islamabad, Islamabad, Pakistan; 7 Addiction Medicine, Department of Psychiatry, Lausanne University Hospital and Lausanne University, Lausanne, Switzerland; 8 Research Centre, University Institute of Mental Health at Montreal and Department of Psychiatry and Addiction Montreal University, Montreal, Canada

**Keywords:** Behavioral addiction, Assessment tools, Sex addiction, Pornography, Internet addiction, “cyberporn”, Nonparaphilia, Impulse control disorder, Internet gaming disorder

## Abstract

**Background:**

Online pornography use, an ever more common activity, has raised myriad psychosocial and clinical concerns. While there is a need to screen for and measure its problematic dimension, there is a debate about the adequacy of existing assessment tools.

**Objective:**

The study compares two instruments for measuring pathological online pornography use (POPU) that are based on different theoretical frameworks—one in line with DSM-5 criteria and the six-component addiction model and one in line with ICD-11 criteria.

**Methods:**

An international sample of 1,823 adults (Mean age = 31.66, SD = 6.74) answered an online questionnaire that included the Short Version of the Problematic Pornography Consumption Scale (PPCS-6) and the Assessment of Criteria for Specific Internet-Use Disorders (ACSID-11). Factorial, correlational, and network analyses were conducted on the data.

**Results:**

Both tools adequately screened for online “addictive” behavior, but the ACSID-11 was superior in assessing the degree of clinical risk.

**Conclusion:**

Depending on the specific aim of the assessment (screening vs. clinical diagnostics), both online pornography measurement tools may be useful.

**Table:** 

Text box 1. Contributions to the literature	
•Among researchers, assessment of Internet-based activities problematic use is a matter of growing theoretical disagreement.	
•Against the tide, in the present study, two assessment instruments developed based respectively on the two main theories of behavioral addictions demonstrate equivalent ability to assess problematic use, among the same sample of participants.	
•In addition, the main findings suggest that de precise adequacy of a given assessment instrument on screening for Internet-based activity problematic use depend on the type of activity and on the studied population.	

## Introduction

The inclusion of Internet Gaming Disorder in the appendix to the 5th edition of the Diagnostic and Statistical Manual of Mental Disorders (DSM-5) [1, 2] and, more recently, of Gaming Disorder in the addictive disorders section of the 11th revision of the International Classification of Diseases (ICD-11) [3, 4] has contributed to a proliferation of scientific research into “new” behavioral addictions—pornography, sports, shopping, social media, dating apps, smartphones, etc. Studies have typically aimed to assess the degree to which increased involvement in “appetitive behaviors” leads to symptoms of an addictive disorder [5–7] or to understand the phenomenological factors associated with, or predictive of, these possible addictions [8–19].

A parallel line of research has aimed to develop instruments for measuring Internet-mediated, potentially addictive, behaviors and help identify pathological cases [10, 20–26] in light of the greater awareness of online risks and concern that around-the-clock access to services that are addictive “by design” may increase problematic use [6, 25, 27–29]. In the case of online pornography, specifically, public health concerns have been raised about the psychological, physical, relational, and societal effects of addictive use [30]. After nearly two decades of “Internet addiction” research that has not always differentiated among specific, potentially addictive, online activities [31, 32], the field can be seen as shifting to exploring unique problematic behaviors—use of online pornography but also that of social media, dating apps, shopping, gambling, and smartphones. Given that personality features such as impulsivity have long been associated with the online experience and problematic online behavior [33], the field may also be seen as moving toward a more nuanced understanding of the psychological drivers of said behaviors.

Still, there is no consensus on when a given potentially problematic online behavior becomes excessive or reaches the threshold of an “addiction” [6, 25, 34], and a growing body of research [6, 35–37] has highlighted the limits of some popular assessment instruments used in making that determination.

### Behavioral addiction models

A behavioral addiction has been defined as a compulsion to engage in a rewarding non-substance-related activity that continues despite negative consequences to the person’s physical, relational, professional, academic, or financial well-being [38]. Similar neural brain reward pathways appear implicated in behavioral and substance addictions [39–41].

Most psychometric tools for assessing potential online behavioral addictions, including pornography, gaming, gambling, or dating apps, are based on the “component model” of behavioral addiction as developed by Griffiths et al. [42] from the model in use for substance addiction [43]. This model assesses addictive behaviors based on six specific components: (a) salience, (b) tolerance, (c) mood modification, (d) relapse, (e) withdrawal, and (f) conflict. This model can be seen to be aligned with the DSM-5 diagnostic criteria for behavioral addictions such as gambling and Internet-based gaming disorder [1, 2].

The component model, however, has been criticized on the grounds that some of its criteria (e.g., salience, tolerance) may not be valid in the context of online activities [6, 44–46]. In addition, the mood modification criterion has been considered too commonly present to correctly discriminate among individuals with or without addictive behaviors [6, 44–46]. Furthermore, high involvement in internet-related behaviors is likely not pathognomonic for addiction [47–48] and may stem from comorbid disorders (e.g., mood disorders, anxiety disorders, attention-deficit and hyperactivity disorder) [49–51].

In a recent study that examined the psychometric validity of the six-component model in the context of social media “addiction”, Fournier et al. [6] concluded that psychometric addiction instruments based on the component model do not form a unitary construct as usually theorized (a global “addiction” score), but are rather characterized by central components (salience and tolerance) and peripheral ones (relapse, mood modification, conflict, and withdrawal) that the instruments “conflate.” This, the authors warn, risks pathologizing normal behavior.

Given the debate around the limitations of the component model and in order to minimize the risk of pathologizing normal online behavior, Muller et al. [25] developed the Assessment of Criteria for Specific Internet-use Disorders (ACSID-11), an instrument based on the ICD-11 [3] criteria for gaming disorder, conceiving of it as “a consistent and economic measure of major types of potential and specific Internet-use disorders” (e.g., pornography, gaming, shopping, gambling, social media) [25, p. 427]. Rather than the six components of the component model, the ACSID-11 is based on the ICD-11 criteria for disorders due to addictive behaviors such as gaming and gambling that have a multifactorial structure [25, p. 430] involving: (a) impaired control over the activity (e.g., onset, frequency, intensity, duration, termination, context); (b) increased priority given to the activity at the expense of other interests and activities; and (c) continuation or escalation of use despite negative consequences [3, 25]. A fourth criterion was added in relation to functional impairment in daily life and marked distress due to the online activity.

In parallel, Bothe et al. [52] developed the Compulsive Sexual Behavior Disorder Scale (CSBDS-19), an ICD-11-based screening tool for measuring compulsive sexual behavior disorder (CSBD), defined as a difficult to resist and persistent tendency to engage in sexual activities despite experiencing significant clinical symptoms and impaired functioning [3]. The CSBDS-19 comprises 5 dimensions: *control* over sexual behavior; *salience* (sexual behavior being the central focus of one’s life); *relapse* (unsuccessful efforts to reduce sexual behavior); *dissatisfaction* (experiencing less or no satisfaction from sexual behavior); and *negative consequences* (sexual behavior generating clinically significant distress or impairment).

The ICD-11-based ACSID and CSBDS are designed to measure the frequency/intensity of the difficult-to-control behavior and its negative consequences [1, 25, 52]. The component model, which is in line with the DSM-5 criteria for behavioral addictions such as gambling and Internet gaming, on the other hand, includes processes (e.g., mood modification) that are considered driving pathways (e.g. “feels better”) [41] and that are included in the DSM-5-based PPCS but not the ACSID or CSBDS.

### Behavioral addiction vs. impulse control disorder

The distinctions between behavior addictions and impulse control disorder have not been clearly delineated, with some disorders having been interchangeably conceptualized and referred to as both. Gambling disorder, for example, was included under the section of “impulse control disorders” in the DSM-IV and under “substance-related and addictive behaviors” in the DSM-5 [53]. Still, attempts to distinguish the two classes have emphasized that behavioral addictions involve a compulsive behavior that is performed repeatedly to reduce emotional or physical discomfort and to alleviate unease or distress [54]. Starting as reward-driven, behavioral addiction processes may soon become dominated by the compulsive element [41]. The impulse control disorder designation, on the other hand, stresses diminished control over an urge-driven act, with little consideration for consequences or despite having already suffered negative effects [54].

Some models consider problematic online activities as impulsive, some as ritualized chronic behaviors reminiscent of the obsessive-compulsive spectrum, and some as a behavioral addiction [55, 56]. Compulsive sexual behavior (CSB), which can be seen to include problematic online pornography use (POPU), has been categorized under impulse control disorders in the ICD-11. There is, however, ongoing debate about whether it might be more closely aligned with addictive behaviors [57–60], especially since limited neurobiological research suggests that it may share characteristics with the addiction model as it has been defined in substance use disorders [60].

### The present study objectives

To inform the debate about the current instruments used in identifying behavioral addictions, this study compared the psychometric properties of the Short Version of the Problematic Pornography Consumption Scale (PPCS-6 [23], designed specifically for online pornography), to the ACSID-11 [25] (applied, in the current study, to online pornography).

The rationale for choosing the PPCS-6 relates to the fact that it is based on the component model, which is widely used for conceptualizing behavioral addictions and is in line with the DSM-5 diagnostic criteria for these conditions [1, 44–46]. The rationale for choosing the ACSID-11 relates to the fact that it is based on the ICD-11 criteria for disorders due to addictive behaviors such as gambling and gaming [3] and because its design allows for assessing a wide range of potentially problematic online activities. Therefore, a comparison between these two instruments also allows for an examination of the extent to which the DSM-5 and the ICD-11 definitions capture the problematic behavior. The choice of problematic online pornography use (POPU) as topic of investigation stems from the fact that online pornography is a highly prevalent behavior and has been studied using both instruments. Indeed, when it comes to the development of the Internet, sex was absolutely foundational. It has been said that lust drives technology, and many everyday online functions, such as secure credit card transactions, videoconferencing, peer-to-peer file sharing, and digital watermarking, had some of their first applications on pornographic sites [61]. Since the arrival of mainstream pornography platforms in the early 2000’s and the rise in smartphone use, online pornography consumption has shown considerable year on year leaps [62–65]. Pornhub, one of the most popular pornographic websites, had approximately 34 billion visits in 2018, 42 billion in 2019 and 55 billion in 2022 [62, 63]. Much has been written about how online pornography consumption may have contributed to decreased satisfaction with “real-life” sexual partners, unrealistic expectations about sexual drive and performance, risky sexual practices, and the emergence of the “hook up” culture [66–69]. Still, most cases of online pornography use are likely non-problematic. Some, however, have been linked to dependence-like states and psychological symptoms [65], with studies of POPU showing prevalence rates between 1 and 15% [65, 70–72].

Given that POPU is one of the most common manifestations of compulsive sexual behavior disorder (CSBD) [73], the CSBD-19 scale [52] was used to test the discriminant validity of the PPCS-6 and the ACSID-11. While all three instruments were designed to measure problematic sexual behavior, it remains unclear how different the constructs assessed by them are or which instrument may best capture POPU. Thus, the present study aims to investigate differences and similarities in the constructs measured by these scales and to assess their respective ability to “capture” POPU.

#### Research questions

Three research questions (RQ) guided the study.

- RQ1: If used in the same study sample, would two instruments with different theoretical backgrounds (the PPCS-6 and ACSID-11) have a similar statistical ability to measure online pornography “addiction”?
- RQ2: Do these scales measure similar constructs?
- RQ3: If we assess “addiction” symptoms using the two scales on the same sample, which symptoms might be central (highly relevant to the “addictive” behavior and might “drive” other symptoms) and which might be peripheral (secondary) in a psychological network analysis?

As this was designed as an exploratory study, no hypothesis associated with these research questions were made.

## Methods

### Participant recruitment and sampling

We recruited 1,823 adults who were active online pornography users to participate in the study by answering an online questionnaire. The inclusion criterion was active online pornography use as determined by self-reported information. The recruitment was conducted anonymously, using the online crowdsourcing platform Prolific [74]. Prolific has been described as having some advantages over other similar platforms, including that it is exclusively dedicated to research studies, and its participants are more ethnically and geographically diverse [75, 76].

### Data collection material

The data was collected via online questionnaire. The questionnaire included sociodemographic questions and several measurement instruments (see descriptive statistics in Table 1), as follows:

**Table 1:** Descriptive statistics of the variables/factors related to the scales used in the present study

Scales’ Dimensions / Total scores	N	Scale	Min-Max	M	SD	Skewness	Kurtosis
**PPCS**							
Salience	1823	1–7	1–7	2.95	1.69	0.52	-0.79
Mood modification	1823	1–7	1–7	4.30	1.75	-0.34	-0.88
Conflict	1823	1–7	1–7	2.27	1.66	1.19	0.33
Tolerance	1823	1–7	1–7	2.56	1.81	0.88	-0.48
Relapse	1823	1–7	1–7	2.70	1.94	0.78	-0.73
Withdrawal.	1823	1–7	1–7	2.05	1.56	1.46	1.15
PPCS total score	1823	1–7	1–7	2.80	1.35	0.71	-0.27
**ACSID-Frequency**							
Impaired control	1823	1–4	1–4	2.06	0.81	0.56	-0.54
Increased priority	1823	1–4	1–4	1.54	0.71	1.38	1.28
Continuation/escalation	1823	1–4	1–4	1.36	0.59	1.91	3.30
Functional impairment	1823	1–4	1–4	1.47	0.67	1.59	2.10
Total score - frequency	1823	1–4	1–4	1.58	0.59	1.41	1.68
**ACSID-Intensity**							
Impaired control	1823	1–4	1–4	1.95	0.83	0.66	-0.45
Increased priority	1823	1–4	1–4	1.51	0.72	1.50	1.55
Continuation/escalation	1823	1–4	1–4	1.32	0.58	2.11	4.19
Functional impairment	1823	1–4	1–4	1.42	0.68	1.74	2.45
Total score - intensity	1823	1–4	1–4	1.52	0.60	1.50	1.78
**ACSID both frequency and intensity combine in one score par dimension**							
Impaired control	1823	1–4	1–4	2.01	0.79	0.61	-0.50
Increased priority	1823	1–4	1–4	1.52	0.69	1.42	1.34
Continuation/escalation	1823	1–4	1–4	1.34	0.57	2.00	3.66
Functional impairment	1823	1–4	1–4	1.44	0.66	1.66	2.25
ACSID overall total score	1823	1–4	1–4	1.55	0.59	0.45	1.74
**Cyberporn use**							
Cyberporn use frequency	1823	2–12	2–12	4.00	1.84	1.14	1.85
**SDI**							
Dyadic	1823	1–8	8–8	5,38	1,26	-0.73	0.87
Solitary	1823	1–8	8–8	5,03	1,58	-0.47	-0.19
**CSBD**							
Control	1823	1–4	1–4	1,65	0.76	1.05	0.21
Salience	1823	1–4	1–4	1,75	0.75	0.80	-0.24
Relapse	1823	1–4	1–4	1,71	0.73	0.95	0.18
Dissatisfaction	1823	1–4	1–4	1,87	0.85	0.66	-0.62
Negative consequences	1823	1–4	1–4	1,55	0.67	1.30	0.92
CSBD-total	1823	1–4	1-3.78	1,67	0.62	0.96	0.17

N = number of participants; M = mean; SD = standard deviation

PPCS = Problematic Pornography Consumption Scale; ACSID = Assessment of Criteria for Specific Internet-use Disorders; SDI = Sexual Desire Inventory; CSBD = Compulsive Sexual Behavior Disorder Scale

When the skewness is between − 0.5 and 0.5, the distribution is fairly symmetric. If the value is greater than + 1, the distribution is right skewed. If the value is less than − 1, the distribution is left skewed. If the Kurtosis value is greater than + 1, the distribution is leptokurtic; if the value is less than − 1, the distribution is platykurtic

Socio-demographic questions covering age, sex, marital status, level of education, and socio-economic status (SES).

The Short Version of the Problematic Pornography Consumption Scale (PPCS-6) [23]. This instrument contains 6 items (e.g., “I felt that porn is an important part of my life”, “I became stressed when something prevented me from watching porn”) and was constructed based on Griffiths’ [42] six-component addiction model covering *salience*, *tolerance*, *mood modification*, *relapse*, *withdrawal*, and *conflict*. Associated with each item is a 7-point Likert response scale, ranging from 1 (Never) to 7 (All the time).

The Assessment of Criteria for Specific Internet-use Disorders questionnaire (ACSID-11) covering frequency and intensity (ACSID-F and ACSID-I, respectively) [25]. The ACSID is a new screening instrument based on ICD-11 criteria for potential Internet-related disorders, including online pornography use, and contains 11 items (e.g., “In the past 12 months, have you tried to stop or restrict the activity and failed with it?”, “In the past 12 months, have you neglected or given up other activities or interests that you used to enjoy because of the activity?”). For each item, there are two questions assessing frequency and intensity (e.g., [a] Think about pornography use - how often? [responses on a scale ranging from 1(“never”) to 4 (“often”)]); [b] Think about pornography use - how intense? [responses on 1–4 points scale from “not at all intense” to “intense”]). The ACSID-11 covers four dimensions: *Impaired control* over the Internet activity (IC, 3 items); *Increased priority* given to the online activity (IP, 3 items); *Continuation/Escalation* (CE, 3 items) of the Internet activity despite negative consequences; *Functional impairment* in daily life (FI, 2 items: functional impairment [FI] and marked distress related to the activity [MD]).

Extent of online pornography use. This was measured using a 2-item scale (“In the last month, how much time have you spent on a typical weekday on pornography?” and “In the last month, how much time have you spent on a typical week-end day on pornography?”), with answers marked on a 6-point scale (1 = Less than half an-hour; 2 = 0.5–1 h; 3 = 1–3 h; 4 = 3–5 h; 5 = 5–7 h; and 6 = More than 7 h). For each participant, the answers on the two items were aggregated into a single total score for extent of online pornography use.

The Sexual Desire Inventory (SDI) [77, 78]. This questionnaire contains 14 items that assess two dimensions of sexual desire: *solitary* (i.e., the desire to engage in sexual behavior alone) and *dyadic* (i.e., the desire to have sexual activity with another person). Using a Likert response scale, participants report on the two dimensions as follows: frequency, from 0 (not at all) to 7 (more than once a day); intensity, from 0 (no desire) to 8 (strong desire); and importance, from 0 (not at all important) to 8 (extremely important). For each participant, two scores were calculated: dyadic sexual desire score and solitary sexual desire score. Higher scores indicate greater sexual desire in each of the two dimensions.

The Compulsive Sexual Behavior Disorder Scale (CSBD-19) [52]. This is an ICD-11-based screening tool for compulsive sexual behavior that is comprised of 19 items (e.g., “Even though my sexual behavior was irresponsible or reckless, I found it difficult to stop”; “My sexual activities interfered with my work and/or education”), each measured according to the five dimensions presented in the introduction section. It must be noted that the CSBD-19 (1–4 points scale) assesses global compulsive sexual behavior, without a specific focus on online pornography.

### Ethics

Participants gave digital informed consent for participating in the study and completing the survey. Participation was voluntary and restricted to those aged ≥ 18. All data was anonymously collected. The survey was conducted in accordance with the Swiss Human Research Act (HRA) [79]. Ethical approval for the research project (no. KB 390/2022) was obtained from The Bioethics Committee of the Nicolaus Copernicus University functioning at Collegium Medicum in Bydgoszcz, Poland.

### Data analysis

First, we conducted descriptive statistics on all study variables (frequencies, means [M], standard deviations [SD], skewness, and kurtosis). These analyses were run with SPSS software statistics (version 29).

To answer RQ1, we conducted several exploratory factorial analyses (EFA) on 70% of the data, including participants’ scores on the six PPCS items/symptoms. These EFA included: unrotated, varimax, and oblimin rotation and eigenvalues vs. parallel analysis to determine the number of factors. Confirmatory factorial analyses (CFA) was conducted on 30% of the PPCS data and on 100% of the data that included participants’ scores on each item of the ACSID-F and ACSID-I. These analyses were carried out to test the dimensionality and construct validity of the two scales.

To evaluate model fit, we used the Comparative Fit Index (CFI), the Tucker-Lewis fit index (TLI), the Standardized Root Mean Square Residual (SRMR), and the Root Mean Square Error of Approximation (RMSEA). Expert statisticians [80, 81] consider that cutoff values of > 0.95 and 0.90 for CFI and TLI, respectively, and of < 0.06 and 0.08 for SRMR and RMSEA, respectively, indicate good or acceptable model fit. A chi-square value divided by degrees of freedom (x^2^/df) of < 3 and of < 5 is another indicator of good or acceptable model fit, respectively [82].

Cronbach alpha coefficient (α) was used as a measure of reliability, with coefficients > 0.8 and > 0.7 indicating good and acceptable internal consistency, respectively [83]. Correlation analyses (Pearson) were used to test convergent validity between different measures of the same or related constructs. According to Cohen [84], a value of *r* = 0.10, 0.30, or 0.50 indicates a small, medium, or large effect, respectively. As the ACSID scale was developed relatively recently by Muller et al. [25] and we wanted to confirm whether our work will yield the same latent structure described by its developers, we did not conduct EFA. However, as the PPCS was developed four years ago [23] and there is some disagreement about whether the six items of the component model [42] on which it was based are unidimensional (one-factor structure) or would better fit within a multifactorial structure, we did conduct EFA. In addition, we conducted binary logistic regression to test the discriminant validity of the PPCS and the ACSID (ability to discriminate between “low clinical risk” [first quartile = coded “0”] vs. “high clinical risk” cases [fourth quartile = coded “1”]).

To answer RQ2, we conducted EFA (using the same parameters as above) on 70% of the data, including participants’ total scores on each of the six PPCS items (salience [S], tolerance [T], relapse [R], conflict [C], mood modification [MM], and withdrawal [W]) and on each of the ACSID-F dimensions (IC, IP, CE, FI). Then, we conducted CFA on 30% of the sample.

It is important to note that varimax rotation returns orthogonal factors, while oblimin allows the factors to not be orthogonal [85]. EFA, correlations, and logistic regression analyses were conducted with SPSS statistics (version 29). CFA were run using Jamovi (version 2.3.26).

To answer RQ3, we conducted a psychological network analysis on the PPCS and ACSID-F symptoms, using the “bootnet” and the “mgm” packages for R. Psychological network analysis was developed in the field of psychopathology [86, 87], although it has also been used in other fields. It belongs to the family of network analyses whose common base is the representation of relations between objects (nodes) using links (edges). In the classic social network analysis, the search focuses on a relational network in which the nodes represent individuals or objects and the links represent social relations. Psychological network analysis is different in that the nodes represent variables (psychological) measured on a sample and the links represent a statistical association among these variables. In health studies, this type of analysis aims to answer three main questions: (a) How does a set of symptoms dynamically relates to other symptoms (partial interactions)?; (b) Which symptoms are the most important (central symptoms that drive the maximum number of other symptoms)?; and (c) Is there cluster and bridges between specific groups of symptoms?

## Results

### Descriptive statistics on the participants sociodemographic variables

The 1823 participants varied in age from 19 to 65 years (M = 31.66, SD = 6.74). Males were more heavily represented than females (male = 1155 [63.4%], female = 636 [34.9%], non-binary = 32 [1.8%]). About half were single and half were married or in a relationship (single = 900 [49.4%], in relationship but not married = 567 [31.1%], married = 317 [17.4%], divorced = 37 [2.0%], widowed = 2 [0.1%]). Socio-economic status varies as follows: low = 483 (26.5%), intermediate = 1265 (69.4%), high = 75 (4.1%). Most of the participants (79.0%) were whites, with other ethnic groups distributed as follows: Asian = 9.4%, mixed-race = 4.8%, other = 2.2%. Educational level (years of schooling) varied from a minimum of 4 years to a maximum of 27 (M = 15.94, SD = 3.08).

Participants resided in the United Kingdom (1,482; 77.2%), the United States (342;17.8%), Ireland (31; 1.6%), Australia (29; 1.5%), Sweden (24; 1.3%), and New Zealand (12; 0.6%). They were of diverse nationalities: 27 European countries (1,466; 76.1%), 2 North-American countries (307; 16%), 14 Asian countries (51; 2.7%), 8 African countries (31; 2%), 2 Oceania countries (34; 1.8%), 8 Latin-American countries (13; 0.8%), and 5 Middle Eastern countries (11; 0.8%).

### Descriptive statistics of responses to scales’ questions, male vs. female comparisons, and correlation between ACSID-F and ACSID-I dimensions

Table 1 shows the descriptive statistics regarding the dimensions and symptoms from all study scales. As shown, only mood modification (a PPCS symptom) and dyadic and solitary sexual desire (the two SDI dimensions) had a score above the middle of the scale range.

The extent of cyberporn use by male participants (M = 2.15, SD = 0.95; scale 1–6) was significantly higher than by female participants (M = 1.75, SD = 0.79), *F*(2, 1788) = 43.19, *p* < 0.001, η^2^_p_ = 0.045. The total PPCS score for male participants (M = 3.08, SD = 1.36; scale 1–7) was also significantly higher than for female participants (M = 2.31, SD = 1.18), *F*(2, 1788) = 75.35, *p* < 0.001, η^2^_p_ = 0.057. The ACSID total score for male participants (M = 1.66, SD = 0.62; scale 1–4) was significantly higher than for female participants (M = 1.36, SD = 0.48), *F*(2, 1788) = 54.91, *p* < 0.001, η^2^_p_ = 0.076. Similarly, the CSBD total score for male participants (M = 1.75, SD = 0.63; scale 1–4) was significantly higher than for female participants (M = 1.56, SD = 0.59), *F*(2, 1788) = 17.88, *p* < 0.001, η^2^_p_ = 0.019.

The PPCS (scale 1–7 points) percentile distribution was as follows: 25th percentile = 1.66; 50th percentile = 2.50; 75th percentile = 3.83. Notably, 19.4% of participants had a score ≥ 4 points, 7.1% had a score ≥ 5 points, and only 1.5% had a score ≥ 6 points. The ACSID (scale 1–4 points) percentile distribution was as follows: 25th percentile = 1.10; 50th percentile = 1.36; 75th percentile = 1.80. Notably, 18.6% of participants had a score ≥ 2 points and only 3.2% had a score ≥ 3 points. The CSBD (scale 1–4 points) percentile distribution was as follows: 25th percentile = 1.15; 50th percentile = 1.47; 75th percentile = 2.05. Notably, 26.1% of participants had a score ≥ 2 points and only 3.7% had a score ≥ 3 points. The chi-square of independence test conducted on the percentiles’ distribution of these three assessment tools indicated significant statistical difference (X^2^ = 43.71, *p* < 0.05, η^2^ = 0.33).

Table 2 shows the correlations between ACSID-F and ACSID-I dimensions. As it can be seen in Table 2, the four ACSID-F factors are highly correlated to the same ACSID-I factors.

**Table 2:** Pearson correlation between symptoms of the assessment of criteria for specific internet-use disorders — frequency and intensity forms

ACSID-F/ACSID-I	Impaired control	Increased priority	Continuation/escalation	Functional impairment	Marked distress
Impaired control	0.87	0.57	0.49	0.53	0.46
Increased priority	0.62	0.90	0.68	0.62	0.55
Continuation/escalation	0.54	0.67	0.91	0.65	0.64
Functional impairment	0.55	0.61	0.63	0.84	0.58
Marked distress	0.50	0.58	0.65	0.63	0.84

ACSID-F = Assessment of Criteria for Specific Internet-use Disorders — Frequency

ACSID-F = Assessment of Criteria for Specific Internet-use Disorders – Intensity

### Comparison of PPCS vs. ACSID psychometric properties (response to RQ1)

#### Assumption for factorial analyses

For the PPCS, the assumptions of adequacy (Kaiser–Meyer–Olkin [KMO] test = 0.864) and sphericity (Bartlett’s test [df = 15] = 5176, *p* < 0.001) for factorial analyses were met [88].

For the ACSID-F and ACSID-I, the assumptions of adequacy (KMO test = 0.906) and sphericity (Bartlett’s test [df = 231] = 39284.45, *p* < 0.001) for factorial analyses were also met [88].

#### EFA results and CFA indices

Regarding the PPCS, EFA based on eigenvalue yielded one factor structure explaining 61% of the variance. EFA based on parallel analysis yielded two different results: the oblimin rotation outputted a structure with two factors explaining 66.3% of the variance (*Factor 1* [salience and mood modification; 41.5%] and *Factor 2* [tolerance, relapse, conflict, withdrawal; 24.08%]); and the varimax rotation outputted a structure with three factors explaining 72% of the variance (*Factor 1* [salience and mood modification; 33.8%], *Factor 2* [tolerance, relapse, conflict; 21.0% ]), and *Factor 3* [withdrawal; 17.4%]).

**Table 3:** The current study confirmatory factorial analysis results: Fit indices by model

Model	x^2^/df	CFI	TLI	SRMR	RMSEA	Number of factors
A	41.11	0.93	0.88	0.051	0.148	1
B	5.42	0.99	0.99	0.011	0.034	2
C	4.87	0.99	0.99	0.046	0.032	3
D	45.75	0.93	0.87	0.050	0.157	2
E	9.753	0.97	0.96	0.036	0.069	4
F	9.473	0.97	0.96	0.034	0.068	4
G	24.06	0.91	0.89	0.055	0.112	2
H	15.02	0.95	0.93	0.036	0.087	4

x^2^/df = Chi-square/degree of freedom; CFI = Comparative Fit Index (CFI); TLI = Tucker-Lewis fit index; RMSEA = Root Mean Square Error of Approximation; SRMR = Standardized Root Mean Square Residual

Model A (61% of the variance) = PPCS conceived as one construct

Model B (66.3% of the variance) = PPCS conceived as two constructs (*Factor 1* [salience and mood modification; 41.5% of variance] and *Factor 2* [tolerance, relapse, conflict, withdrawal; 24.08% of variance])

Model C (72.2% of the variance) = PPCS conceived as three constructs (*Factor 1* [salience and mood modification; 33.8% of variance], *Factor 2* [tolerance, relapse, conflict; 221.0% of variance]), and *Factor 3* [withdrawal; 17.4%])

Model D (Fournier et al., 2022) = PPCS conceived as two constructs (*Factor 1* [salience and tolerance] and *Factor 2*[mood modification, relapse, conflict, withdrawal])

Model E = ACSID-F.

Model F = ACSID-I.

Model G (66.29% of the variance) = ACSID-F and PPCS conceived as two constructs (all ACSID-F four dimensions [ 35.49% of the variance] and all PPCS six dimensions [30.8% of the variance])

Model H (66.4% of the variance) = ACSID-I and PPCS conceived as four constructs (Factor 1 [ACSID-Increased priority, ACSID-Continuation/escalation, ACSID- Functional impairment; 24.57% of the variance], Factor 2 [PPCS-Salience and PPCS-Mood modification; 23.88% of variance], Factor 3 [PPCS-Tolerance, PPCS-Relapse, PPCS-Conflict, PPCS-Withdrawal; 12.72% of the variance], Factor 4 [ACSID-Impaired Control; 5.19% of the variance])

PPCS = Problematic Pornography Consumption Scale. ACSID = Assessment of Criteria for Specific Internet-use Disorders; PPCS = Problematic Pornography Consumption Scale

Table 3 represents the results of all CFA conducted. As shown, regarding the PPCS scale, the model with one factor had poor adjustment to the data (Model-A: x^2^/df = 41.11; CFI = 0.93; TLI = 0.88; SRMR = 0.051; RMSEA = 0.148), whereas the model with two factors (Model-B) and the model with three factors (Model-C) showed relatively good fit. Overall, Model-C seemed to be the best model (x^2^/df = 4.87; CFI = 0.99; TLI = 0.99; SRMR = 0.046; RMSEA = 0.032). Interestingly, Model-B differs from the two-factor model found by Fournier et al. [6] in an Italian sample, which had: *Factor 1* (salience and tolerance) and *Factor 2* (relapse, conflict, mood modification, withdrawal), with the following CFA fit indices: x^2^ = 98.729, df = 8, *p* < 0.001; CFI = 0.986; TLI = 0.974; SRMR = 0.073; RMSEA = 0.033. Therefore, using the Fournier et al. [6] factorial structure above, we conducted CFA on the present study data (Model-D, see Table 3) and found fit indices that indicated relatively poor adjustment to the data: x^2^/df = 45.75; CFI = 0.93; TLI = 0.87; SRMR = 0.050; RMSEA = 0.157.

The CFA conducted on the ACSID-F data (Model-E) suggested good adjustment: x^2^/df = 9.75; CFI = 0.97; TLI = 0.96; SRMR = 0.036; RMSEA = 0.069. The CFA conducted on the ACSID-I data (Model-F) indicated good fit: x^2^/df = 9.47; CFI = 0.97; TLI = 0.96; SRMR = 0.034; RMSEA = 0.068.

#### Internal reliability

The α of the PPCS one-factor structure (all six items/symptoms) was: 0.87. The α of the PPCS two-factor structure was: *Factor 1* (salience and mood modification) = 0.76 and *Factor 2* (tolerance, relapse, conflict, withdrawal) = 0.87.

The α of the ACSID-F was 0.90, and the α of the ACSID-I was 0.92. The α of the ACSID-F factorial structure was: Impaired control (IC) = 0.78; Increased priority (IP) = 0.85, continuation/escalation (CE) = 0.78, and functional impairment (FI) = 0.77. The α of the ACSID-I factorial structure was: IC = 0.80; IP = 0.86, CE = 0.79, and FI = 0.78.

#### Convergent validity

Table 4 shows the correlations between the six PPCS symptom scores, the PPCS total score, participants’ pornography use extent, sexual desire (dyadic and solitary), and the five factors of the CSBD. All *r* values are positive and show medium to high effect sizes.

**Table 4:** Pearson bivariate correlation between the symptom from Problematic Pornography Consumption Scale and from Assessment of Criteria for Specific Internet-use Disorders, sexual desire, and the participants socio-demographic characteristics

Factors / Variables	Extent of online pornography use	SDI Dyadic	SDI Solitary	CSBD Control	CSBD Salience	CSBD Relapse	CSBD Dissatisfaction	CSBD Negative consequences	CSBD Total score	Age	Sex^#^	Edu-level	SES
**PPCS**													
Salience	0.39	0.30	0.51	0.35	0.40	0.38	0.20	0.30	0.38	0.17	-0.18^**^	-0.03	-0.01
Mood modification	0.34	0.34	0.52	0.31	0.33	0.34	0.18	0.28	0.34	0.09	-0.21^**^	-0.03	-0.05
Conflict	0.37	0.20	0.33	0.51	0.42	0.46	0.27	0.52	0.53	0.01	-0.21^**^	-0.04	-0.05
Tolerance	0.40	0.23	0.35	0.52	0.46	0.52	0.32	0.52	0.57	0.03	-0.20^**^	-0.04	-0.02
Relapse	0.33	0.21	0.31	0.47	0.38	0.53	0.26	0.44	0.50	0.03	-0.27^**^	-0.02	0.02
Withdrawal.	0.35	0.19	0.29	0.52	0.44	0.49	0.31	0.53	0.56	0.00	-0.15^**^	-0.05	-0.04
S-MM score	0.41	0.36	0.57	0.37	0.40	0.40	0.21	0.32	0.40	0.14	-0.22^**^	-0.04	-0.03
C-T-R-W score	0.43	0.25	0.38	0.60	0.50	0.59	0.34	0.59	0.64	0.02	-0.25^**^	-0.04	-0.02
PPCS total score	0.47	0.32	0.49	0.57	0.52	0.59	0.33	0.55	0.62	0.07	-0.27^**^	-0.05	-0.03
**ACSID-Frequency**													
Impaired control	0.41	0.21	0.32	0.43	0.31	0.43	0.24	0.42	0.44	-0.06	-0.24^**^	-0.01	0.01
Increased priority	0.42	0.17	0.30	0.51	0.43	0.47	0.29	0.53	0.55	0.04	-0.18^**^	-0.01	-0.01
Continuation/escalation	0.31	0.13	0.19	0.45	0.33	0.41	0.27	0.50	0.49	-0.01	-0.17^**^	-0.00	0.02
Functional impairment	0.36	0.13	0.24	0.48	0.35	0.42	0.25	0.50	0.49	-0.06	-0.19^**^	-0.01	-0.01
Total score - frequency	0.44	0.18	0.31	0.54	0.41	0.50	0.30	0.57	0.57	-0.03	-0.23^**^	-0.01	-0.00
**ACSID-Intensity**													
Impaired control	0.41	0.22	0.32	0.45	0.34	0.45	0.24	0.45	0.47	-0.03	-0.24^**^	-0.01	0.02
Increased priority	0.41	0.19	0.29	0.50	0.43	0.48	0.28	0.52	0.54	0.06	-0.18^**^	-0.03	-0.01
Continuation/escalation	0.32	0.13	0.20	0.43	0.33	0.39	0.26	0.49	0.47	0.02	-0.15^**^	-0.01	0.02
Functional impairment	0.36	0.13	0.23	0.47	0.35	0.41	0.26	0.49	0.49	-0.02	-0.17^**^	-0.01	0.00
Total score - intensity	0.43	0.19	0.30	0.54	0.42	0.50	0.30	0.56	0.57	-0.00	-0.21^**^	-0.02	0.00
**ACSID both frequency and intensity combine in one score par dimension**													
Impaired control	0.43	0.22	0.33	0.45	0.34	0.45	0.25	0.45	0.47	-0.04	-0.25^**^	-0.01	0.01
Increased priority	0.43	0.18	0.30	0.52	0.44	0.49	0.29	0.54	0.56	0.05	-0.19^**^	-0.02	-0.01
Continuation/escalation	0.32	0.13	0.20	0.45	0.34	0.41	0.27	0.51	0.49	0.00	-0.16^**^	-0.00	0.02
Functional impairment	0.37	0.13	0.24	0.49	0.36	0.43	0.26	0.51	0.51	-0.04	-0.18^**^	-0.01	-0.00
ACSID overall total score	0.44	0.19	0.31	0.55	0.42	0.51	0.31	0.57	0.58	-0.01	-0.23^**^	-0.01	0.003

^#^Bacause Sex is nominal variable (1 = male; 2 = female) we conducted zero-order person correlation analysis. The negative sign preceding the values means that female had statistically lower scores than male participants

**Correlation is significant at the 0.01 level; * Correlation is significant at the 0.05 level

PPCS = Problematic Pornography Consumption Scale; ACSID = Assessment of Criteria for Specific Internet-use Disorders; CSBD = Compulsive Sexual Behavior Disorder Scale; SDI = Sexual Desire Inventory; Edu-level = education level; SES = socio-economic status

Table 4 also shows the correlations between, on the one hand, ACSID-F and ACSID-I factorial scores and ACSID-F and ACSID-I total scores, and, on the other hand, participants’ online pornography use extent, sexual desire (dyadic and solitary), and the five factors of the CSBD scale. Most, but not all, *r* values were positive and of medium to high effect sizes.

Importantly, as can be seen in Table 4, the values of the ACSID-F and ACSID-I correlations with the extent of online pornography use, SDI, and CSBD dimensions are generally lower (although not in a statistically significant manner), compared with the values of the correlations between PPCS and the online pornography use, SDI, and CSBD variables.

#### Discriminant validity

Table 5 displays the results of the logistics regression models (Model 1–4). They represent a discriminant analysis testing. It shows:

**Table 5:** The current logistic regression results: Estimated beta coefficients of the associations between the dimensions/symptoms from the Problematic Pornography Consumption Scale and the Assessment of Criteria for Specific Internet-use Disorders and compulsive sexual behavior “low clinical risk” vs. “high clinical risk” cases

Latent classes	Covariates	b	SE	*p*	OR	OR 95%CI	
**Model 1: Nagelkerke R**^**2**^ **= 60%**							
Scales total score	PPCS	1.085	0.102	< 0.001	2.96	2.42	3.61
	ACSID	1.357	0.230	< 0.001	3.88	2.47	6.09
**Model 2 : Nagelkerke R**^**2**^ **= 59%**							
PPCS symptoms: the unidimensional model	Salience	0.097	0.074	0.190	1.10	0.95	1.27
	Mood modification	0.075	0.068	0.271	1.07	0.94	1.23
	Conflict	0.175	0.081	0.031	1.19	1.01	1.39
	Tolerance	0.348	0.081	< 0.001	1.41	1.20	1.66
	Relapse	0.268	0.062	< 0.001	1.30	1.15	1.47
	Withdrawal	0.505	0.101	< 0.001	1.65	1.35	2.02
**Model 3 : Nagelkerke R**^**2**^ **= 57%**							
PPCS total scores by factor: the bidimensional model	S-MM score	0.168	0.073	0.022	1.18	1.02	1.36
	C-T-R-W score	1.254	0.095	< 0.001	3.50	2.90	4.22
**Model 4: Nagelkerke R**^**2**^ **= 50%**							
ACSID total score by factor	Impaired control	0.677	0.138	< 0.001	1.96	1.50	2.58
	Increased priority	1.255	0.219	< 0.001	3.50	2.28	5.39
	Continuation/escalation	0.307	0.275	0.265	1.35	0.79	2.33
	Functional impairment	0.666	0.234	0.004	1.94	1.23	3.08

*b* = beta coefficient; SE = standard error; *p* = p-value at 0.05 level; OR = odds-ratio; CI = confidence interval

PPCS = Problematic Pornography Consumption Scale. S-MM = salience and mood modification total score; C-T-R-W = conflict, tolerance, relapse, and withdrawal total score. ACSID = Assessment of Criteria for Specific Internet-use Disorders– both Frequency and Intensity score aggregate. CSBD = Compulsive Sexual Behavior Disorder Scale

1. To what extent the participants’ scores on the PPCS and ACSID symptoms/factors discriminated between (a) participants with low CSBD total-scores (fist quartile = “low clinical risk” [coded “0”]) and (b) participants with high CSBD scores (fourth quartile = “high clinical risk” [coded “1”]);
2. To what extent participants’ total scores on PPCS and ACSID-F/ACSID-I discriminated between (a) participants with low CSBD total-scores and (b) participants with high CSBD total-scores.

The “low clinical risk” group (*n* = 499) had a CSBD total-scores ≤ 1.16. Among them, 275 were male, 210 were female, and 14 were non-binary. The “high clinical risk” group (*n* = 446) had a CSBD total-scores ≥ 2.05. Among them, 323 were male, 118 were female, and 5 were non-binary. There was a statistical difference between the number of males and females present in each group (X^2^[df = 2] = 31.05, *p* < 0.001).

The descriptive statistics for the CSBD score were as follows: scale = 1–4 points; min score = 1, max score = 3.79, mean = 1.68, median = 1.47. The percentiles were: 25% = 1.16, 50% = 1.47, 75% = 2.05. The number of participants by quartile were: first quartile = 499 (27.4%), second quartile = 425 (23.3%), third quartile = 453 (24.8%), fourth quartile = 446 (24.5%).

The omnibus test model coefficients for Model 1 (PPCS total score and ACSID total score; X^2^ (df = 2) = 564.87, *p* < 0.001; Nagelkerke R^2^ = 60%), Model 2 (each of the PPCS symptoms score; X^2^ (df = 6) = 551.71, *p* < 0.001; R^2^ = 59%), Model 3 (each of the two PPCS factors total score; X^2^ (df = 2) = 545.99, *p* < 0.001; R^2^ = 57%), and Model 4 (each of the four ACSID factors total score; X^2^ (df = 4) = 441.36, *p* < 0.001; R^2^ = 50%) were significant, indicating a good fit to the data [89].

Model 1 results (see Table 5) suggests that the ACSID scale (odds-ratio [OR] = 3.88) performed better than the PPCS scale (OR = 2.92) at discriminating between “low clinical risk” of CSBD and “high clinical risk” of CSBD. Among the PPCS six symptoms (see Model 3), withdrawal (OR = 1.65), tolerance (OR = 1.41), and relapse (OR = 1.30) were the dimensions most able to discriminate between participants with “low clinical risk” and “high clinical risk” of CSBD. Among the ACSID four factors (see Model 2), increased priority (OR = 3.50) was the dimension that was the most able to discriminate between participants with “low clinical risk” and “high clinical risk” of CSBD.

The R^2^ values indicate the percentage of change in the dependent variable (CSBD) explained by the predictor variables in the model. The OR can be read here as the “effect-size” associated with each predictor in the model.

### EFA results (response to RQ2)

For the EFA on the PPCS and ACSID dimensions total scores, assumptions of adequacy (KMO = 0.919) and sphericity (Bartlett’s [df = 120] = 11692.43, *p* < 0.001) were met [88]. The α were: ACSID-dimensions-total-score = 0.88 and PPCS = 0.87.

The EFA based on eigenvalue (including rotation) yielded a two-factor structure explaining 66.29% of the variance (*Factor 1* [all ACSID-F five dimensions; 35.49%], *Factor 2* [all PPCS six dimensions; 30.8%]). The EFA based on parallel analysis and varimax rotation yielded a four-factor structure explaining 66.4% of the variance (*Factor 1* [ACSID-Increased priority, ACSID-Continuation/escalation, ACSID-Functional impairment, ACSID-Marked distress; 24.57%], *Factor 2* [PPCS-Salience and PPCS-Mood modification; 23.88%], *Factor 3* [PPCS-Tolerance, PPCS-Relapse, PPCS-Conflict, PPCS-Withdrawal; 12.72%], *Factor 4* [ACSID-Impaired control; 5.19%]).

The CFA conducted on the above mentioned two-factor structure (Table 3, Model-G) showed a relatively poor fit to the data: x^2^/df = 24.06; CFI = 0.91; TLI = 0.89; SRMR = 0.055; RMSEA = 0.112. The CFA conducted on the above mentioned four-factor structure (Table 3, Model-H) showed acceptable adjustment to the data: x^2^/df = 15.02; CFI = 0.95; TLI = 0.93; SRMR = 0.036; RMSEA = 0.087.

### Network relationships between symptoms (response to RQ3)

#### Full zero-order Pearson correlations between the symptoms

Table 6 displays the correlations between all symptom score values. As shown, all correlations were medium or large. However, the correlations were stronger (e.g., *r* > or = 0.50) within the same category of symptoms. Among symptoms belonging to the two different assessment tools, the correlations were stronger between: ACISD-IP x PPCS-C = 0.63; ACSID-IC x PPCS-*R* = 0.59; ACSID-IP x PPCS-W = 0.59; ACSID-IP x PPCS-T = 0.57; ACCSID-IF x PPCS-C = 52; and ACSID-IC x PPCS-C = 0.50.

**Table 6:** Correlations between symptoms of Problematic Pornography Consumption Scale and the symptoms of Assessment of Criteria for Specific Internet-use Disorders -- Frequency form

SYMPTOMS	PPCS-S	PPCS-MM	PPCS-C	PPCS-T	PPCS-R	PPCS-W	ACSID-IC	ACSID-IP	ACSID-CE	ACSID-FI	ACSID-MD
PPCS-S	1.00										
PPCS-MM	0.61	1.00									
PPCS-C	0.47	0.42	1.00								
PPCS-T	0.52	0.48	0.68	1.00							
PPCS-R	0.41	0.41	0.57	0.63	1.00						
PPCS-W	0.47	0.39	0.64	0.65	0.58	1.00					
ACSID-IC	0.36	0.40	0.50	0.51	0.59	0.44	1.00				
ACSID-IP	0.43	0.35	0.63	0.57	0.49	0.59	0.60	1.00			
ACSID-CE	0.29	0.23	0.48	0.44	0.43	0.49	0.52	0.70	1.00		
ACSID-FI	0.35	0.30	0.52	0.49	0.46	0.46	0.55	0.63	0.66	1.00	
ACSID-MD	0.24	0.21	0.45	0.42	0.41	0.43	0.51	0.60	0.67	0.63	1.00

PPCS = Problematic Pornography Consumption Scale

ACSID = Assessment of Criteria for Specific Internet-use Disorders

PPCS-S = Salience, PPCS-MM = Mood modification, PPCS-C = Conflict, PPCS-T = Tolerance, PPCS-R = Relapse, PPCS-W = Withdrawal.

ACSID-IC = Impaired control, ACSID-IP = Increased priority given to the activity, ACSID-CE = Continuation/escalation of use despite negative consequences, ACSID-FI = Functional impairment in daily life, ACSID-MD = Marked distress

Correlation analyses (Pearson) were used to test convergent validity between different measures of the same or related constructs

These analyses were run with IBM SPSS statistics (version 29). According to Cohen (1988), a value of *r* = 0.10, 0.30, 0.50 indicates a small, medium, large effect, respectively

Cohen, J. (1988). Statistical power analysis for the behavioral sciences (2nd ed.). New York: Academic Press

#### Symptoms network relationships

The stability metrics of this network were as follows: strength coefficient = 0.72, expected influence coefficient = 0.75, and edge coefficient = 0.75. All these indices indicate that the model had excellent stability [86, 87].

**Fig. 1:**
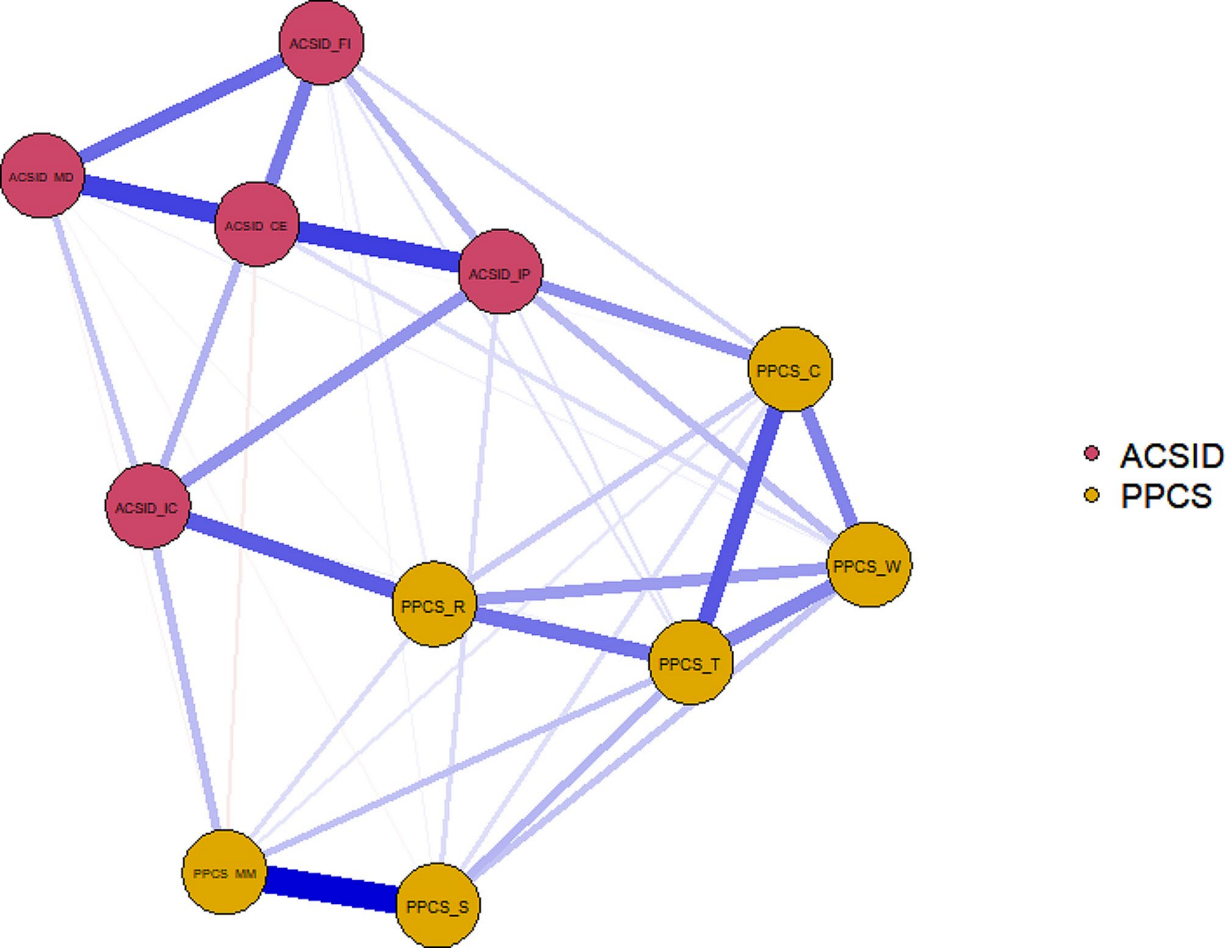
The problematic pornography use variables structural and interactional network: all-sample. Circles are called “nodes” and they represent each variable (which in psychological network analysis are called “symptoms”) included in the model. The lines are called “edges” and they indicate the association between the symptoms and represent the standardized partial correlations. Blue edges indicate positive correlations, while orange edges indicate negative correlations. Thicker edges indicate larger partial correlations PPCS = Problematic Pornography Consumption Scale. PPCS-S = Salience, PPCS-MM = mood modification, PPCS-C = conflict, PPCS-T = tolerance, PPCS-R = relapse, PPCS-W = withdrawal ACSID = Assessment of Criteria for Specific Internet-use Disorders. ACSID-IC = Impaired control, ACSID-IP = Increased priority given to the activity, ACSID-CE = Continuation/escalation of use despite negative consequences, ACSID-FI = Functional impairment in daily life; ACSID-MD = Marked distress

Figure 1 shows the results of the psychological network analysis. The circles are referred to as “nodes” and represent each variable “symptom” included in the model. The lines are referred to as “edges” and represent the standardized partial correlations among symptoms (Spearman correlations, in this case). Blue edges indicate positive correlations, whereas orange edges (if any) indicate negative correlations. Thicker edges indicate larger partial correlations.

**Fig. 2:**
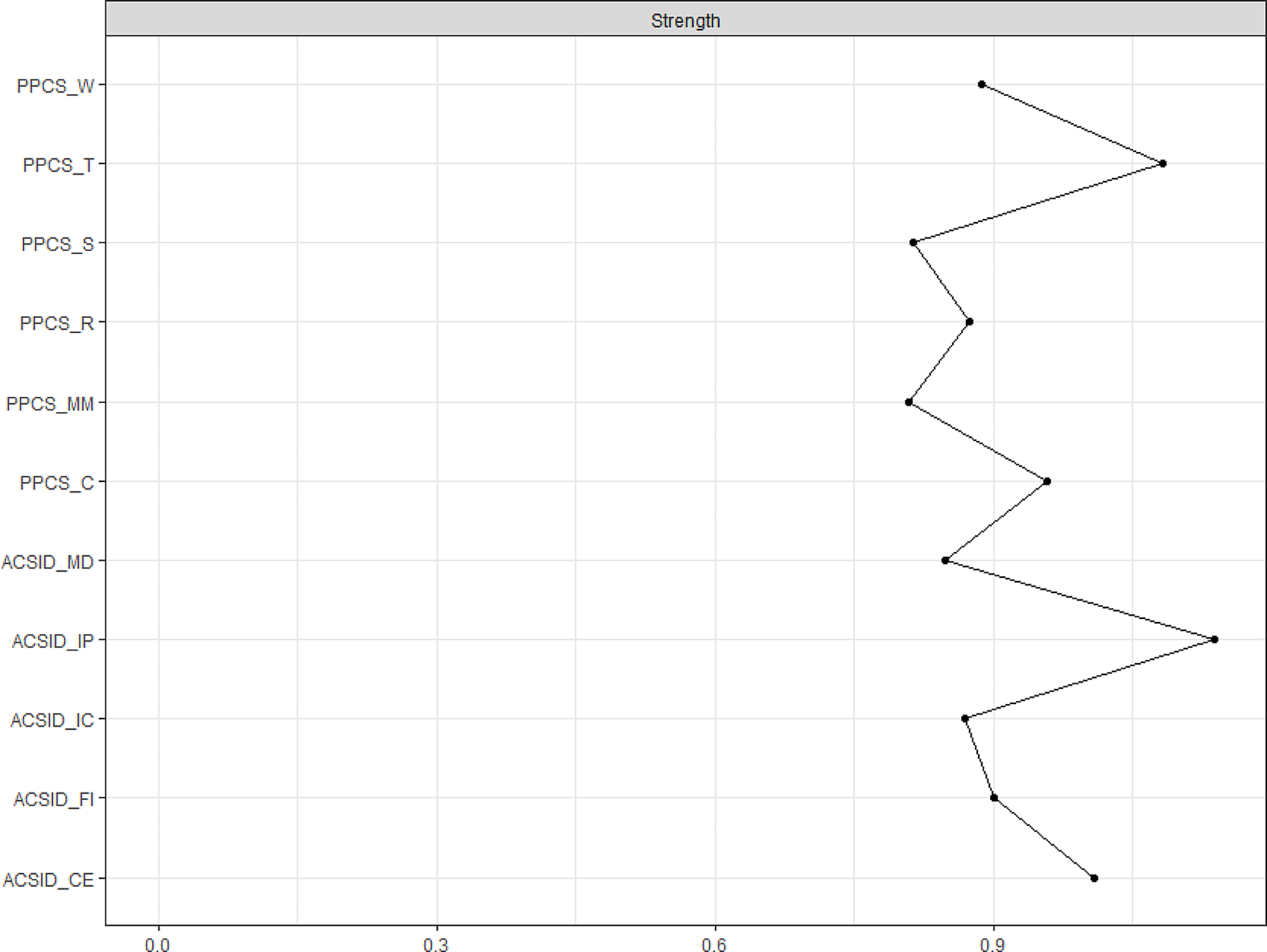
Strength centrality of the modeled symptoms: all-sample. The symptoms are in the vertical axe. The Strength Centrality (SC) values are in horizontal axe PPCS = Problematic Pornography Consumption Scale. PPCS-S = Salience, PPCS-MM = mood modification, PPCS-C = conflict, PPCS-T = tolerance, PPCS-R = relapse, PPCS-W = withdrawal ACSID = Assessment of Criteria for Specific Internet-use Disorders. ACSID-IC = Impaired control, ACSID-IP = Increased priority given to the activity, ACSID-CE = Continuation/escalation of use despite negative consequences, ACSID-FI = Functional impairment in daily life; ACSID-MD = Marked distress

Figure 2 shows the centrality strength of ACSID and PPCS symptoms. The vertical axis represents symptoms, and the horizontal axis the corresponding centrality strength values. As seen, the central symptoms (the two most extreme-right line peaks) are ACSID-IP (SC = 1.95) and PPCS-T (SC = 1.44). Furthermore, there is a bridge pathway between the two categories of symptoms (ACSID and PPCS) that goes from ACSID-IC to PPCS-R and vice-versa.

## Discussion

The present study explored the very common behavior of online pornography as an example of a potentially addictive Internet activity and compared two theoretically different instruments (PPCS vs. ACSID) to inform the debate surrounding the adequacy of current Internet-based pornography and other behavioral addiction assessment tools.

### PPCS-ACSID: descriptive results and male vs. female comparison

With very few exceptions (three variables: mood modification [from PPCS symptoms], dyadic and solitary sexual desire [from SDI dimensions]), the results from the measurement tools indicate that the mean values are below the midpoint of the respective scale. This suggests that POPU is a relatively marginal phenomenon in the studied sample of online pornography users. Similar results have been found in previous studies [12, 90].

While this study was not designed to assess POPU or CSBD prevalence, it might be relevant to note that 19.4%, 18.6%, and 26.1% of participants had a total score equal or greater than the midpoint of the PPCS, ACSID, and CSBD scales, respectively. In addition, only 1.5%, 3.2%, and 3.7% of participants had a scores within the higher end of the PPCS (≥ 6 points), ACSID (≥ 3 points), and CSBD (≥ 3 points) scales. The prevalence of POPU and CSBD is highly disputed in the literature, in part because of the diversity of instruments used to assess them, the difficulty in agreeing on cuff-off values, and doubts about sample representativeness [65]. The 1.5%, 3.2%, and 3.7% prevalence rates in our study represent a conservative cut-off and fall within the 1–15% prevalence range for online pornography “addiction” as reported by several studies published in the last 10 years [65, 70–72].

Among online pornography users, extent of use, “addictive” use (as indicated by total PPCS and ACSID scores), and “compulsive” sexual behavior disorder (as indicated by total CSBD score) were all higher among male participants compared to female participants. Similar results have been found in other studies [12, 14, 23, 25, 52]. Several factors may help explain this: higher levels of testosterone in males, the hormone described as driving sexual desire [91]; genetic differences in vulnerability to the development and maintenance of this behavior [92]; sex-based differences in the neurophysiological aspects of sexual behavior [93]; differential impact on relationships and sexual and psychosocial functioning [94]; and cultural norms and social expectations that lead women to underreport sexual activity compared to men [95].

### PPCS: Unifactorial or multifactorial?

As in the study by Fournier et al. [6], the EFA and CFA conducted on the present data indicate that the PPCS is a multifactorial measurement instrument (structured around at least two latent constructs). This finding is in contradiction with the unifactorial component model of behavioral addiction [42] on which the PPCS was based, and in contradiction with previous studies [23, 96, 97]. There is, however, an important difference between our findings and those of Fournier at al. [23]. While in both studies a structure with two factors was found, the two dimensions’ content was relatively different. In our study, the first dimension was composed of the salience and mood modification PPCS symptoms; the second, of the conflict, tolerance, relapse, and withdrawal PPCS symptoms. In the study by Fournier at al. [23], the first factor included salience and tolerance; the second, the remaining symptoms. Such differences in factor composition were frequently noted in studies related to the assessment of Internet-related addictive behaviors and have been hypothesized to be due to differences in sample characteristics and the type of Internet activity being studied [98]. Thus, the current difference may stem from the fact that our study focused on online pornography and used a more diverse sample (North America, Europe, and South Pacific), whereas Fournier et al. [23] focused on social media use in a sample drawn exclusively from Italy. Furthermore, this difference suggests that scales based on the component model may be associated with less reproducibility in terms of latent structure and screening or diagnostic capabilities across population samples and various potentially addictive behavior.

Based on their findings, Fournier at al. [23] claimed that screening tools based on the component model [38] may lead to pathologizing mere involvement in appetitive behaviors, because some of the six components (i.e., salience, tolerance) may be inadequate for assessing psychopathological symptoms. The current study results did not confirm this, given that (a) a different factorial structure was found and (b) tolerance was among the symptoms that were more able to discriminate between low clinical risk and high clinical risk cases of CSBD. Thus, these two studies’ relative “contradiction” suggests that the ability of assessment tools based on the component model to screen for addictive behavior may vary based on the Internet-related activity or on sample characteristics.

### PPCS-ACSID: psychometric properties comparison (response to RQ1)

Regarding internal reliability, the PPCS had a α that varied from 0.76 to 0.87, and the ACSID had a α that varied from 0.78 to 0.92. While this represents a small advantage for the latter, both measurement tools had rather similar internal reliability metrics.

As for convergent validity, the correlation values between the two scales and the other modeled variables (extent of online pornography use, SDI, and CSBD dimensions) suggest small advantage for the PPCS. Conversely, the findings indicate that ACSID significantly outperforms the PPCS in discriminating between individuals with “low clinical risk” vs. “high clinical risk” behavior. This finding suggests that the ICD-11-based ACSID-11 is better than the component model and DSM-5-based PPCS at identifying severe cases, and therefore may reduce the over-pathologizing risk. However, this remains a hypothesis that should be tested more thoroughly in future studies.

Overall, the results suggest that both the PPCS and the ACSID-11 can appropriately assess “addictive” online pornography, although the ACSID-11 may have superior discriminative properties. The present finding is, however, limited by the fact that the Compulsive Sexual Behavior Disorder Scale (CSBD-19) [52] used in our analysis is not specific to online pornography.

### PPCS-ACSID: same or different constructs (response to RQ2)

Findings from the present study indicate that when assessing a problematic online behavior, assessment tools based on different theoretical models such as PPCS (DSM-5 criteria, component model) and the ACSID-11 (ICD-11 criteria) do not measure the same constructs. Indeed, the CFA yielded a factorial structure that clearly separated PPCS symptoms on one side and ACSID symptoms on another. This suggests that the two measurement tools are not interchangeable and that the most discriminant criteria from the PPCS (i.e., tolerance) are not strongly associated with the ones driven by the ASCID-11 (i.e., increased priority). Another indication that these two instruments may not assess the same construct stems from the statistically significant difference in the calculated prevalence of “at risk” POPU (1.5% [PPCS] and 3.2% [ACSID]). In this respect, the ACSID prevalence of “at risk” participants is much closer to that of the CSBD “at risk” prevalence (respectively 3.2% [ACSID] and 3.7% [CSBD], not statistically significant). Of note, like the ACSID, the CSBD was designed as an ICD-11-based screening measure.

### PPCS-ACSID: central symptoms (response to RQ3)

Overall, network analysis results indicate that relationships are stronger within the symptoms of each scale, suggesting a strong independence for each of the two scales and a distinct “raison d’être”, further supporting the idea discussed above of relatively different constructs. However, a “bridge” exists between the symptoms of the two scales—namely between the *impaired control* symptom (ACSID) and the *relapse* symptom (PPCS). This result would seem to make sense given that relapse (“when I vowed not to watch porn anymore, I could only do it for a short period of time”) can be seen as a facet of impaired control [99, 100].

Further, within the whole network, there are two central symptoms, one for each scale—*increased priority* (ACSID; SC = 1.95) and *tolerance* (PPCS; SC = 1.44). According to psychological network analysis theory [86, 87], central symptoms drive others and are crucial to the screening of the target addictive behavior, whereas other symptoms are more “peripheral” to the assessment. The fact that there is at least one central symptom per scale confirms the relevance of each. If all central symptoms belonged to one scale, it would suggest dominance of that scale over the other.

The fact that increased priority (IP) was found to be the most important central symptom is in line with a growing consensus in addiction studies around it as a core symptom of addictive disorders [34]. The present findings also add to the evidence that tolerance plays an important role in addictive pornography [101] and behavioral addictions overall [102]. Indeed, there is some evidence that the arousal effects associated with pornography are particularly sensitive to tolerance [103]. Still, there seems to be less consensus regarding tolerance as a criterion for assessing addictive behaviors than for IP [101, 102, 104].

In addition, the fact that IP is a central symptom may suggest that it is related to stronger addiction driving paths (e.g., “feels better” and “must do”) and is therefore involved in the shift from reward-driven to compulsive-driven processes and behaviors [41]. Overall, these findings suggest that IP is not only a core characteristic of addictive behaviors, but may develop based on driving factors (cravings, desires, negative reinforcement, feelings of compulsive motivations) referred to in the description of clinical features in the ICD-11 and in several theoretical models [41], including the Interaction of Person-Affect-Cognition-Execution (I-PACE) model of Internet-use disorders [105].

### Limitations

The methodology used for data collection does not allow us to know how representative the study sample is of the population of online pornography users. Also, the lack of information on sexual orientation or sexual interests may have homogenized distinct groups within the population of online pornography users. In addition, it is possible that, if they had been tested against the “gold-standard” comprehensive clinical interview, the scales’ diagnostic validity, prognostic value and overall clinical utility might have been different.

### Future studies

Building on the present findings and given our study limitations, future research should examine the comparative psychometric properties of these two types of instruments in representative samples to test which can better discriminate “normal” from “pathological” online pornography use, as well as other potentially problematic Internet-related behaviors. To advance the debate around behavioral addictions and impulse control disorders, it would be informative to collect data on clinical populations using screening tools based on different theoretical models and to conduct psychological network analyses to see if similar or different results can be found with respect to the centrality of impulsive or addictive symptoms. Finally, studies that compare the gold-standard clinical interview based on ICD-11 criteria to that based on DSM-5 criteria in the same sample and for the same potentially problematic online activity would be particularly illuminating.

## Conclusion

The results of the present study suggest that two different Internet-based activities problematic use screening tools, based respectively on the DSM-5 criteria and ICD-11 criteria, can both adequately screen for “addictive” behavior, although the ACSID-11 is likely to have superior discriminant capabilities for assessing low clinical risk vs. high clinical risk. Tools based on the component model of behavioral addiction such as PPCS seem more suitable for use in general population whereas those based on ICD-11 criteria such as ACSID seem suitable both for non-clinical and clinical purposes, such as identifying individual “at risk” who may be targets for a psycho-educational intervention.

## Data Availability

The questionnaires and the data used in this study are available at: https://gitlab.huma-num.fr/gveracruz/behavioral-addiction-assessment/-/tree/main/Data.

## Abbreviations

DSM-5: The 5th version of Diagnostic and Statistical Manual of Mental Disorders
ICD-11: The 11th revision of the International Classification of Diseases
WHO: World Health Organization
PPCS: Problematic Pornography Consumption Scale
PPCS-S: Salience
PPCS-MM: Mood modification
PPCS-C: Conflict
PPCS-T: Tolerance
PPCS-R: Relapse
PPCS-W: Withdrawal
ACSID-F/I: Assessment of Criteria for Specific Internet-use Disorders– Frequency/Intensity
ACSID-IC: Impaired control
ACSID-IP: Increased priority given to the activity
ACSID-CE: Continuation/escalation of use despite negative consequences
ACSID_FI: Functional impairment in daily life
ACSID-MD: Marked distress
CSBD: Compulsive Sexual Behavior Disorder Scale
CSBD-C: Control (failure to)
CSBD-S: Salience
CSBD-R: Relapse
CSBD-D: Dissatisfaction
CSBD-NC: Negative consequences (distress or impairment)
SES: Socio-economic status
EFA: Exploratory factorial analysis
PCA: Principal component analysis
CFA: Confirmatory Factorial Analysis
I-PACE: Interaction of Person-Affect-Cognition-Execution
